# Herbivore range expansion triggers adaptation in a subsequently-associated third trophic level species and shared microbial symbionts

**DOI:** 10.1038/s41598-019-46742-3

**Published:** 2019-07-16

**Authors:** Fushi Ke, Shijun You, Sumei Huang, Weijun Chen, Tiansheng Liu, Weiyi He, Dandan Xie, Qiang Li, Xijian Lin, Liette Vasseur, Geoff M. Gurr, Minsheng You

**Affiliations:** 1 State Key Laboratory of Ecological Pest Control for Fujian and Taiwan Crops, Institute of Applied Ecology, Fujian Agriculture and Forestry University, Fuzhou, 350002 China; 2 Joint International Research Laboratory of Ecological Pest Control, Ministry of Education, Fuzhou, 350002 China; 3 Key Laboratory of Integrated Pest Management for Fujian-Taiwan Crops, Ministry of Agriculture, Fuzhou, 350002 China; 4 Department of Biological Sciences, Brock University, 1812 Sir Isaac Brock Way, St. Catharines, Ontario, L2S 3A1 Canada; 5 Graham Centre, Charles Sturt University, Orange, NSW 2800 Australia

**Keywords:** Ecology, Evolution

## Abstract

Invasive species may change the life history strategies, distribution, genetic configuration and trophic interactions of native species. The diamondback moth, *Plutella xylostella* L., is an invasive herbivore attacking cultivated and wild brassica plants worldwide. Here we present phylogeographic analyses of *P. xylostella* and one of its major parasitoids, *Cotesia vestalis*, using mitochondrial markers, revealing the genetic diversity and evolutionary history of these two species. We find evidence that *C. vestalis* originated in Southwest China, then adapted to *P. xylostella* as a new host by ecological sorting as *P. xylostella* expanded its geographic range into this region. Associated with the expansion of *P. xylostella*, *Wolbachia* symbionts were introduced into local populations of the parasitoid through horizontal transfer from its newly associated host. Insights into the evolutionary history and phylogeographic system of the herbivore and its parasitoid provide an important basis for better understanding the impacts of biological invasion on genetic configuration of local species.

## Introduction

Human activities and climate change have allowed many plant and animal species to recently expand their geographic ranges, a phenomenon likely to continue despite increasing quarantine efforts^1^. Impacts of biological invasions manifest at scales ranging from individuals to ecosystems and landscapes, potentially leading to evolutionary changes^2,3^. In the course of species’ expansion, the population dynamics and evolutionary processes of local flora and fauna may be affected^4–6^. Many factors, including genetic structure and variation in local populations, determine the capacity of native species to interact and adapt to the invader^5^. Higher trophic level species, such as parasitoids, in a given location may switch among taxonomically disparate but ecologically similar types of hosts by ecological sorting^7^, resulting in new associations or assemblages of related species^8^.

The diamondback moth, *Plutella xylostella* L. (Lepidoptera: Plutellidae), is a brassica-specialist herbivore of global significance^9–12^. It has achieved wide distribution across the world (i.e. East Asia and Oceania) in recent centuries, most likely due to human activities, such as globalization of trade and brassica crop cultivation^10,13,14^. In recent studies of *P. xylostella*, genetic homogeneity has been found in many populations across Asia-Pacific regions^15–17^. Insect movement due to air currents and transportation of agricultural products have been proposed as the main reasons for high levels of gene flow for *P. xylostella*^15,16,18–20^.

A broad range of natural enemies, including parasitoids, arthropod predators, pathogenic fungi and bacteria, attack *P. xylostella*^9–11^. Among these, *Cotesia vestalis* (=*plutellae*) Haliday (Hymenoptera: Braconidae) is one of its most important biocontrol agents^10,11,21^ occurring in 38 countries^10^. Whilst *C. vestalis* has been introduced to Australia, North America, and the Caribbean in over 20 classical biological control programs^10,22^, there are no records of it being introduced to Japan, Vietnam, Malaysia (Cameron Highlands) and China^22–25^. Yet, *C. vestalis* is reported to be among the most predominant parasitoids of *P. xylostella* across parts of East Asia^25–27^, most likely associated to its tolerance to high temperatures^28^ and insecticides^25,26^, and suggesting it may be native to this region.

The evolutionary success and diversification of insects has been aided, to some extent, by their complex associations with microorganisms^29,30^. Over the recent decades, *Wolbachia* symbionts have attracted intensive research effort due to their diverse behavioral effects in a broad range of insect hosts, ability to manipulate the host reproductive system, and potential role in biological control of pests^31^. *Wolbachia* are generally assumed to be maternally inheritable, with vertical transfer from egg cytoplasm to offspring, though recent studies have demonstrated horizontal transfer from infected to uninfected species^32^.

Invasive species may change distribution, genetic configuration and trophic interactions of other native species and trigger rapid adaptation^33,34^, which is increasingly becoming a major focus of research in evolutionary biology. In the present study, using samples of *P. xylostella* and *C. vestalis* collected from five Asian countries of China, Nepal, Thailand, Malaysia and Vietnam, we used a set of mitochondrial genes to analyze the phylogeographic relationships to (1) reveal the genetic diversity and demographic history of the two species; (2) identify the geographic origin of *C. vestalis*; and (3) address the adaptation of *C. vestalis* to the invasive *P. xylostella* in this region. Considering the *Wolbachia-*arthropod associations and the potential role in intra-specific interactions, we also investigated the impacts of horizontal transfer of *Wolbachia* on the genetic configuration and co-evolution of local assemblages.

## Results

### Genetic diversity

A total of 1,621 bp DNA was obtained from concatenation of three *P. xylostella* mitochondrial genes (hereafter referred as p3m). From 323 *P. xylostella* individuals coming from 29 sampling locations (Table 1), we found 187 polymorphic sites and 212 haplotypes, representing a high haplotype diversity with an average of 0.931. We identified 174 haplotypes represented by single individuals, with the remainders represented by multiple *P. xylostella*. Nucleotide diversity was overall low with an average of 0.329%, except for the samples from three locations of Quanzhou in China (FJQZ, 0.606%), Cameron Highlands in Malaysia (MLCH, 0.906%) and Katmandu in Nepal (NPKT, 0.682%) (Table 1).

**Table 1:** Parameters of Genetic Diversity and Demographic History of the *P. xylostella* and *C. vestalis* Populations Based on Three Mitochondrial Genes (*CoxI*, *Cytb* and *NadhI*).

Population	n	L	S	h	Hd	*θ*(%)	Tajima’s *D*	Fu’s *Fs*
XJSHZ	4|1	1621|1232	11|0	4|1	−|−	−|−	−|−	|−
JLCC	18|14	1621|1232	45|6	16|2	0.987|0.143	0.421|0.070	−1.976*|−1.959*	−7.859**|2.207
LNSY	17|21	1621|1232	26|9	17|4	1.000|0.614	0.285|0.278	−1.613|1.258	−15.584*** |3.870
GSJQ	5|1	1621|1232	7|0	5|1	1.000|−	0.185|−	−0.747 |−	−2.238|−
BJ	8|4	1621|1232	20|1	8|2	1.000|−	0.383|−	−1.036|−	−3.319*|−
TJ	4|19	1621|1232	8|2	4|2	−|0.105	−|0.017	−|−1.511*	−|0.021
NX	1|2	1621|1232	0|3	1|2	−|−	−|−	−|−	−|−
SDQD	18|13	1621|1232	47|7	13|3	0.928|0.564	0.410|0.237	−2.108*|1.122	−3.216*|3.671
HNZZ	20|20	1621|1232	49|5	17|4	0.984|0.284	0.462|0.041	−1.842*|−1.974**	−7.260**|−1.565
SXSL	8|2	1621|1232	6|0	3|1	0.607|−	0.115|−	−0.920|−	1.412|−
AHHF	8|4	1621|1232	17|0	8|1	1.000|−	0.322|−	−1.028|−	−3.771*|−
SH	15|19	1621|1232	31|0	14|1	0.990|−	0.338|−	−1.790|−	−8.319 ***|−
CQ	7|	1621|	9|	4|	0.714|	0.170|	−1.319 |	0.495 |
HBWH	16|10	1621|1232	30|7	15|3	0.992|0.378	0.314|0.114	−1.807*|−1.839 *	−10.052 ***| 1.160
SCLZ	|8	|1232	|2	|2	|0.250	|0.041	|−1.310	| 0.762
JXNC	14|14	1621|1232	24|4	12|4	0.978|0.396	0.283|0.046	−1.657 *|−1.798*	−5.995**|−1.640
GZGY	17|10	1621|1232	47|25	16|5	0.993|0.667	0.470|0.431	−1.931*|−1.899**	−8.345***|1.728
FJFZ	13|19	1621|1232	17|6	12|2	0.987|0.199	0.210|0.097	−1.758|−0.988	−8.828***|3.392
FJPT	8|3	1621|1232	16|0	8|1	1.000|−	0.291|−	−1.213|−	−4.09*|−
FJQZ	11|10	1621|1232	36|7	8|3	0.945|0.378	0.606|0.114	−0.940|−1.839*	0.216 |1.160
FJXM	7|14	1621|1232	3|6	3|2	0.524|0.363	0.065|0.177	−0.654|0.550	0.110|4.962*
YNYX	15|13	1621|1232	43|23	14|6	0.990| 0.769	0.464|0.795	−1.903 *|1.389	−6.435**| 3.524
FJZZ	7|3	1621|1232	18|3	7|1	1.000|−	0.376|−	−0.952 |−	−2.550*|−
GDGZ	17|17	1621|1232	25|0	15|1	0.985|−	0.270|−	−1.736|−	−9.920***|−
GXNN	14|19	1621|1232	23|20	12|4	0.978|0.380	0.285|0.328	−1.528|−1.126	−5.970**|4.333*
NPKT	7|17	1621|1232	34|5	7|5	1.000|0.426	0.682|0.056	−1.168|−1.719*	−1.386|−2.308
TLPH	7|10	1621|1232	15|0	6|1	0.952|−	0.294|−	−1.228|−	−1.228|−
VTDL	14|14	1621|1232	26|0	14|1	1.000|−	0.313|−	−1.613|−	−10.580***|−
MLKK	12|10	1621|1232	14|2	7|3	0.773|0.511	0.209|0.054	−1.143|−0.184	−1.028|−0.272
MLCH	11|13	1621|1232	35|1	7|2	0.909|0.282	0.906|0.023	0.912|−0.274	−2.451|0.240

Note: n represents the *P. xylostella*|*C. vestalis* sampled individuals, L is the length of DNA fragments, S is the segregating sites, h and Hd are the number and diversity of haplotypes, θ is nucleotide diversity, “−” denotes populations with <5 individuals or with only one haplotype and they were not used for calculation of population parameters, “|” symbolizes the separation of *P. xylostella* (left) and *C. vestalis* (right), the significance of statistic tests are indicated by “*” (*P* < 0.05), “**” (*P* < 0.01) and “***” (*P* < 0.001). The first 25 populations listed are from China, with the other five populations from Nepal (1), Thailand (1), Vietnam (1) and Malaysia (2). The samples within a country are listed in order from the most northerly to the most southerly latitude. Full information of the populations refers to Table S1.

For *C. vestalis*, based on 1,232 bp DNA from three mitochondrial genes (hereafter referred as c3m), we found a relatively low haplotype and nucleotide diversity with an average of 0.415 and 0.172%, respectively. From 324 individuals, 43 polymorphic loci and 29 haplotypes were identified, with 19 haplotypes coming separately from single individuals and the rest from multiple individuals. We also observed higher haplotype and nucleotide diversity from two sampling locations of Guiyang (GZGY: Hd = 0.667 and *θ* = 0.431%) and Yuxi (YNYX: Hd = 0.769 and *θ* = 0.795%) in Southwest China (Table 1).

### Phylogeny and haplotype network

**Figure 1:**
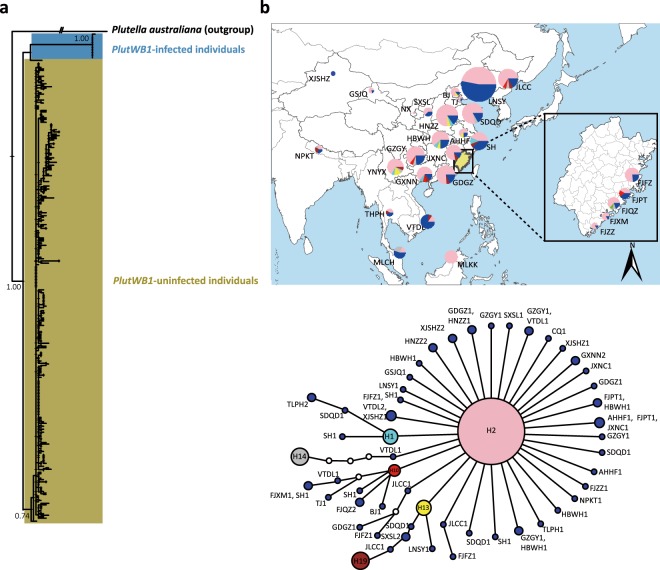
Phylogenetic Tree and Haplotype Network of *Plutella xylostella*. (**a**) Phylogeny of *P. xylostella* based on the concatenated *CoxI*, *Cytb* and *NadhI* genes using maximum likelihood algorithm with 1000 bootstraps, with *P. australiana* as an outgroup. (**b**) Haplotype network based on *Cytb* for *P. xylostella*. Haplotypes with frequency ≤4 are illustrated in blue and labeled with sampling location acronyms and numbers; small empty circles represent unsampled haplotypes. Numbers upon branches are bootstrap values > 0.5.*  PlutWB1*: a specific Wolbachia strain previously identified in *P. xylostella*.

**Figure 2:**
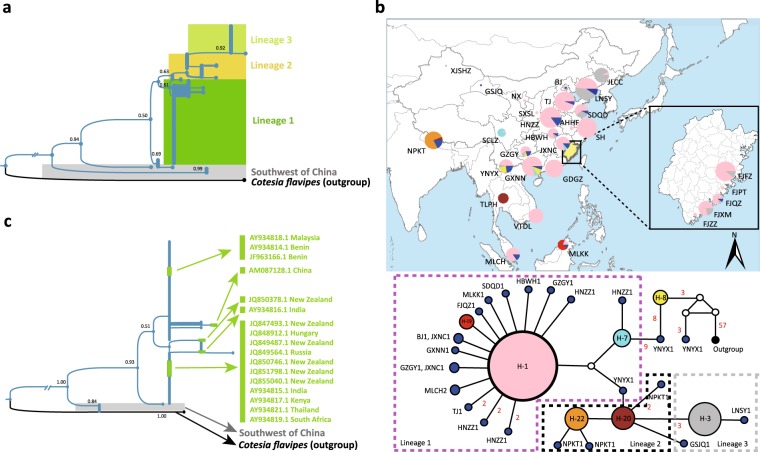
Phylogenetic Tree and Haplotype Network of *Cotesia vestalis*. (**a**) Phylogeny of *C. vestalis* based on the concatenated *CoxI*, *Cytb* and *NadhI* genes using maximum likelihood algorithm with 1000 bootstraps, with *C. flavipes* as an outgroup. (**b**) Haplotype network based on three concatenated genes, *CoxI*, *Cytb* and *NadhI* for *C. vestalis*. The number of mutations >1 is presented beside the corresponding branches; haplotypes with frequency ≤4 are illustrated in blue and labeled with sampling location acronyms and numbers; small empty circles represent unsampled haplotypes; haplotypes labeled YNYX are from Southwest China. (**c**) Phylogeny of global *C. vestalis* samples based on the *CoxI* gene (545 bp) using maximum likelihood algorithm with 1000 bootstraps, with *C. flavipes* as an outgroup. Individuals in green indicate recruited individuals from Europe, Africa, Oceania and Asia. Numbers upon branches are bootstrap values > 0.5.

The p3m-based phylogenetic tree revealed overall low genetic differentiation among individuals in terms of the branch length, and no isolated clusters containing individuals from specific geographic regions or populations, regardless of high genetic differentiation identified between Wolbachia *PlutWB1*-infected and -unfected individuals (Fig. 1a). The *Cytb*-based network exhibited a star-like shape with many unique haplotypes present at the terminals (Fig. 1b), indicating recent population expansion by *P. xylostella*. In terms of this *Cytb*-based network, haplotype 2 (H2) was dominant and present in most sampled populations. The frequency of identified haplotypes was randomly distributed in each of the *P. xylostella* populations. The c3m-based phylogenetic tree (Fig. 2a) and haplotype network (Fig. 2b) showed four separate lineages with multiple basal clusters/lineages containing the samples from Southwest China. Lineage 1 comprised the samples from part of China, Malaysia and Vietnam, Lineage 2 consisted of individuals from China, Thailand and Nepal, while lineage 3 was represented by individuals from China only. Using the *CoxI* gene sequences of our *C. vestalis* samples and additional sequences from India, Kenya, Benin, Hungary, Malaysia, New Zealand and Russia, we constructed a *CoxI*-based phylogeny (Fig. 2c) that does not change the overall 3cm-based topology of the basal position and paraphyly of samples from Southwest of China (Fig. 2a,b).

**Figure 3:**
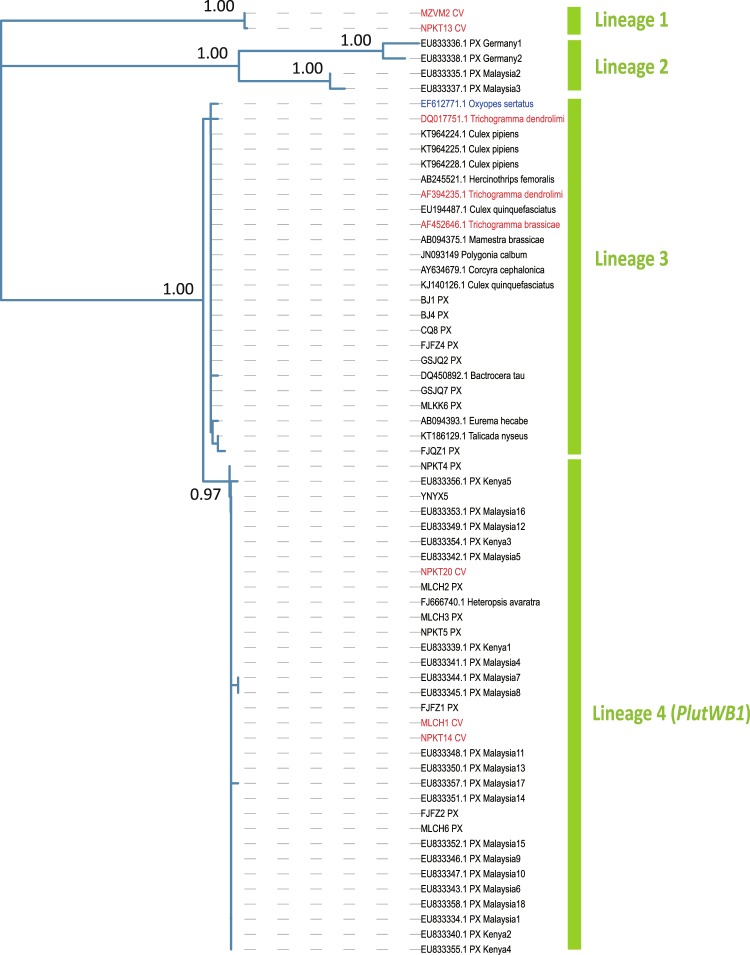
The *Wsp*-based Phylogenetic Tree of *Wolbachia*. The gene sequences are coloured for different hosts (black: herbivore; red: parasitoid; blue: predator). PX = *P. xylostella*; CV = *C. vestalis*. Numbers upon branches are bootstrap values > 0.5.

Four primary lineages were present on the *Wsp*-based Phylogeny of *Wolbachia* (Fig. 3). Hosts involved in lineage 1 and 2 are *C. vestalis* (Lineage 1) and *P. xylostella* (Lineage 2), respectively, while lineage 4 was identified in both of these two species (Fig. 3).

### Demographic history

**Figure 4:**
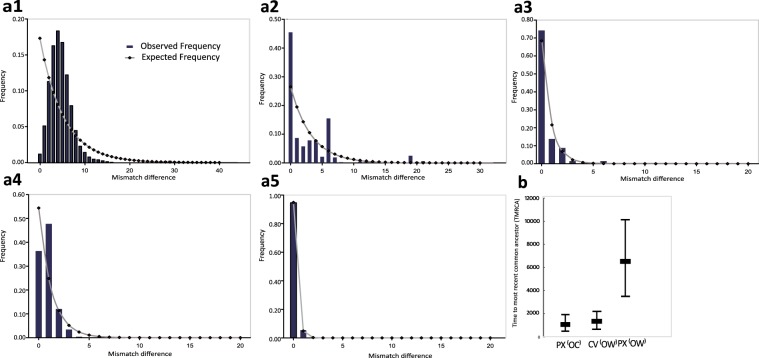
Demographic Inference. (**a1**–**a5**) Mismatch distributions of *P. xylostella* and *C. vestalis* based on three concatenated genes, *CoxI*, *Cytb* and *NadhI* (**a1**) *P. xylostella* with all sampled individuals; (**a2**) *C. vestalis* with all sampled individuals; (**a3**) Lineage 1 of *C*. v*estalis*; (**a4**) Lineage 2 of *C*. v*estalis*; and (**a5**) Lineage 3 of *C*. v*estalis*). (**b**) Estimation of the *CoxI-*based TMRCA for *P. xylostella* and *C. vestalis*. PX: *P. xylostella*; CV: *C. vestalis*; OC: Oceania; OW: Old World.

Neutrality tests for *P. xylostella* were conducted using Tajima’s *D* and Fu’s *Fs* statistics (Table 1). The p3m-based Tajima’s *D* and Fu’s *Fs* statistics were significantly negative (Tajima’s *D* = −2.501, *P* < 0.001; Fu’s *Fs* = −5.532, *P* < 0.05) when all sampled populations were considered as one group. A significantly negative Tajima’ *D* value was associated with a significantly negative Fu’s *Fs* value, suggePhysting a recent expansion of *P. xylostella* populations in this region. The p3m-based mismatch distribution was unimodal when all sampled individuals of *P. xylostella* were considered as one group for analysis (Fig. 4a1), further supporting the concept of a recent expansion of the *P. xylostella* populations in East Asia.

Based on the neutrality tests for *C. vestalis*, c3m-based Tajima’s *D* and Fu’s *Fs* statistics also had significantly negative values (Tajima’s *D* = −1.747, *P* < 0.05; Fu’s *Fs* = −10.475, *P* < 0.001) when all sampled individuals taken as one group. In the defined clusters, only Lineage 1 showed significantly negative values of Tajima’s *D* (*D* = −2.323, *P* < 0.01) and Fu’s *Fs* (*Fs* = −23.426, *P* < 0.001), suggesting a recent expansion event of the *C. vestalis* populations. No population expansion events could be inferred in Lineage 2 (Tajima’s *D* = −1.421, *P* > 0.05; and Fu’s *Fs* = −3.066, (0.01 < *P* < 0.05)) and Lineage 3 (Tajima’s *D* = −1.133, *P* > 0.05; and Fu’s *Fs* = −1.362, *P* > 0.05). The c3m-based mismatch distribution was multimodal when all sampled individuals were considered as one group, but the three defined lineages exhibited unimodal distributions (Fig. 4a2–a5).

## Discussion

The paraphyly nature of samples from Southwest China, located at the early branching nodes of both c3m-based (Fig. 2a) and *CoxI*-based (Fig. 2c) phylogeny, suggests that Southwest China is the geographical origin of *C. vestalis*. Such an inference was also supported by the high nucleotide polymorphism of *C. vestalis* populations (YNYX and GZGY) in Southwest China (Table 1). We also found that all our sampled *C. vestalis* individuals derived from Southwest China based on the c3m-based phylogenetic tree (Fig. 2a) and haplotype network (Fig. 2b). According to the *CoxI*-based global phylogeny of *C. vestalis* (Fig. 2c, and supported by c3m tree, Fig. 2b), *C. vestalis* individuals from African, Oceanian and European countries were evolutionarily closely related to those from Thailand (TLPH) and Nepal (NPKT).

*C. vestalis* is one of the most common parasitic wasps of *P. xylostella*^10^ and previously considered to be native in Malaysia^24^, Japan^23^, and China^25^. It has also been suggested to originate from Europe based on the species description using Ukrainian specimens^21^. However, our *Cox I*-based phylogenetic analysis demonstrated that *C. vestalis* populations in Europe, Africa and Oceania derived from East Asia (Fig. 2b). Although no samples from the New World were included in this study, we speculate that the haplotypes from the New World may derive from the haplotypes of the Old World as *C. vestalis* was reported to be recently introduced into North America^22,35^ and South America^21,35^ as a biological control agent.

*P. xylostella* was recorded to colonize many regions (including East Asia) of the world in recent centuries^14,15^. A significantly negative Tajima’ *D* value was associated with a significantly negative Fu’s *Fs* value, suggesting a recent expansion of *P. xylostella* populations in East Asia, which is further supported by the unimodal p3m-based mismatch distribution of all sampled individuals. Our metadata analysis revealed a regional expansion of *C. vestalis* populations associated with the invasion and colonization of *P. xylostella* in East Asia. A comparable observation has also been reported for two *Diadegma* parasitoids of *P. xylostella* in Europe^36^. The results of BEAST analysis also showed that the most recent common ancestor (TMRCA) of *C. vestalis* was between the TMRCAs of *P. xylostella* in Old World (OW) and Oceania (OC), suggesting that the population expansion of *C. vestalis* could be related to the regional invasion of *P. xylostella* into Oceania through East Asia. Based on our results, we propose that the regional distribution of *C. vestalis* is a case of ecological sorting^7^ that add *P. xylostella*, which had become the dominant herbivore in brassica crops of East Asia^11,37^, to the hosts for *C. vestalis*^38^. Such a new trophic association may facilitate the rapid adaptation of *C. vestalis*, by the previously-documented means of altered the life history traits and physiological manipulation^39,40^, to *P. xylostella*.

The co-occurrence of *plutWB1*^41^ in *P. xylostella* and *C. vestalis* (Lineage 4 in Fig. 3) suggested horizontal transfer of *Wolbachia* between herbivore and parasitoid. Our observation of *Wolbachia*-infected *P. xylostella* pupae and adults implied that the direction of this horizontal transfer was from *P. xylostella* to *C. vestalis*, given that *C. vestalis* would kill *P. xylostella* before pupation^35^. In addition, in concordance with findings of Delgado & Cook^41^, a distinct clade of five individuals (from different sampling locations) infected by *plutWB1* were identified in the phylogenetic tree of *P. xylostella* (Fig. 1a), suggesting a long history of co-evolution between *P. xylostella* and this *Wolbachia* strain and a horizontal transfer scenario from the herbivore to its parasitoid. Another distinct clade (Lineage 3) in the *wsp*-based phylogenetic tree showed that the host of *Wolbachia* involved parasitoids, herbivores and predators, suggesting that the horizontal transfer of *Wolbachia* can occur across multiple trophic levels.

Li *et al*.^42^ reported that *Bemisia tabaci*-associated *Wolbachia* can be horizontally transferred between infected and uninfected individuals via plants. This can be further supported by a recent study of flowers and wild megachilid bees, in which the plants can act as hubs for bacterial transmission between multiple organisms^43^. In the present study, using our *wsp* sequences and the NCBI-based *wsp* sequences, we found that the individuals infected by *Wolbachia* were involved with several species of herbivores (Lineage 3 in Fig. 3). We assumed that the presence of such a *Wolbachia* lineage (Lineage 3) in various herbivores might have come from food intake or potentially from horizontal transfer between the species.

**Figure 5:**
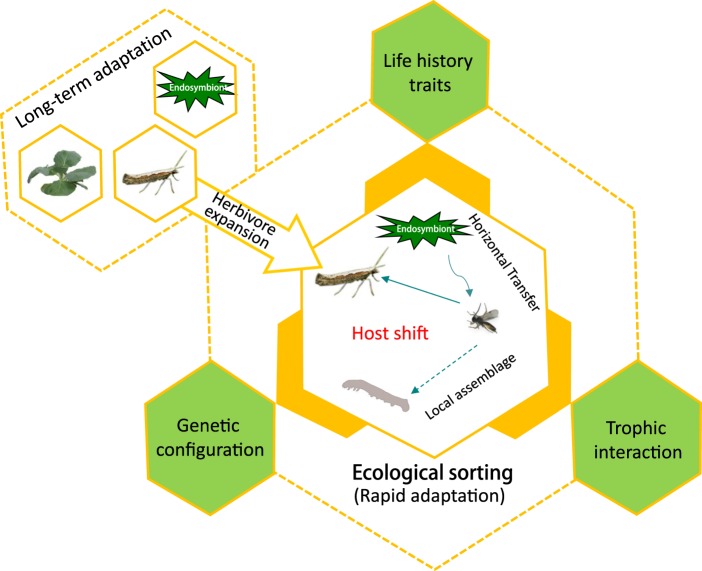
A Schematic Map Illustrating the Rapid Adaptation of *C. vestalis* to the Invaded *P. xylostella* (as a new host) through the Ecological Sorting Process.

In this study, using an interactive system involving an invasive herbivore, *P. xylostella*, and its parasitoid, *C. vestalis*, we demonstrated how the invasion of an alien host herbivore (*P. xylostella*) could significantly affect the genetic variation of a higher trophic species (*C. vestalis*). Through ecological sorting, this parasitoid could have switched from an original local host to *P. xylostella* and, as an important agent of classical biological control, it underwent significant, human-aided, population expansion. In addition, during this expansion, the endosymbiont *Wolbachia* (*plutWB1*) in *P. xylosetlla* was also introduced into local species. Our work provides a comprehensive picture (Fig. 5) of how invasion by an alien species can trigger significant evolutionary changes in a newly associated parasitoid and the transmission of exotic bacteria into local species, leading to the formation of new biological interactions and the genetic configuration of local species.

## Methods

### Sample collection and species identification

We collected *P. xylostella* and *C. vestalis* samples from the same or nearby cabbage and broccoli fields in China (25), Nepal (1), Thailand (1), Vietnam (1) and Malaysia (2) from 2012 to 2014 (Fig. S1; Table S1). Twenty-eight samples of *P. xylostella* and *C. vestalis* were collected from common locations, and one sample of *P. xylostella* was collected in Chongqing, China (CQ) and its counterpart *C. vestalis* sample from nearby, Luzhou in Sichuan Province, China (SCLZ) (Fig. S1; Table S1). The second and third instar larvae of *P. xylostella* were maintained on vegetable leaves for emergence of parasitoids. *P. xylostella* pupae and adults, and *C. vestalis* cocoon*s* were morphologically identified and preserved in 95% ethanol. Specimens were stored at −80 °C prior to DNA extraction. A total of 323 *P. xylostella* and 324 *C. vestalis* individuals were used in this study. We used a 600 bp mitochondrial gene sequence (*CoxI*) (Table S2) and DNA barcoding criteria to individually confirm the species identity of *P. xylostella* and *C. vestalis* based on BOLD^44^.

### DNA Extraction and sequencing

Total genomic DNA was extracted from individual insects using DNeasy Blood and Tissue Kit (Qiagen, Germany). Three mitochondrial genes, *CoxI*, cytochrome b (*Ctyb*), and NADH dehydrogenase subunit I (*NadhI*) were sequenced for both *P. xylostella* and *C. vestalis* (Table S2). Primers were developed for *Cytb* and *NadhI* of *P. xylostella* as well as *Cytb* and *NadhI* of *C. vestalis* using Primer Premier version 5 (Premier Biosoft International, Palo Alto, CA, USA) based on the reference mitochondrial genomes. Primers of other gene segments were referred to published references (Table S2).

PCR was conducted using the Mastercycler pro system (Eppendorf, Germany) under the following conditions: an initial denaturation for 2 min at 94 °C, followed by 35 cycles of 10 s at 96 °C, 15 s at specific annealing temperature of each genes (Tables S2), and 1 min at 72 °C, and a subsequent final extension for 10 min at 72 °C. Amplified products were purified and bidirectionally sequenced using the ABI 3730xl DNA Analyzer by Sanboyuanzhi Biotechnology Co., Ltd. (Beijing, China). All the sequences were deposited in Genebank database (accession number from KX604356 to KX606864).

Infection of *P. xylostella* and *C. vestalis* by *Wolbachia* was determined using ∼600 bp products of the *wsp* gene amplified with specific primers (Table S2). A positive control of PCR reaction (with DNA of *Wolbachia* infected samples as templates) was used to test infection of *Wolbachia* in samples.

### Genetic analysis

Sequences for each of the gene fragments were aligned using MEGA5.2^45^. All mitochondrial sequences for each of the individuals of both insect species were aligned independently using MAFFT-7.037^46^. Conservative regions selected by Gblock-0.91b^47^ were used for gene concatenation, which was performed by Sequence-Matrix-1.7.8 with default parameters^48^. Parameters of genetic diversity, haplotype diversity (Hd) and nucleotide diversity (***θ***), were calculated using the DnaSPv5^49^. Populations with <5 individuals (3 populations of *P. xylostella* and 12 of *C. vestalis*) were not included in the calculation of the parameters related to the genetic diversity (Table 1).

### Phylogenetic and network analysis

Phylogeographic analysis can be used to explore the evolutionary history of a species^50–52^ or to examine the temporal and spatial effects on co-evolutionary relationships of closely related species^53,54^. This type of study can help reveal the impacts of biological expansions on local communities over wide spatial scales. Using the sequences of three concatenated mitochondrial genes, phylogenetic relationships were constructed for *P. xylostella* (here after p3m) and *C. vestalis* (here after c3m). We selected *Cotesia flapvis*, which is from the *Cotesia* genus, as the outgroup for *C. vestalis* phylogenetic tree construction. The *CoxI* sequences of *C. vestalis* and *C. flapvis* (outgroup) from NCBI (http://www.ncbi.nlm.nih. gov/) were also downloaded for construction of a global phylogenetic tree of *C. vestalis*. The phylogeny of *wsp* gene was developed using the NCBI-based *wsp* sequences with the best hit (with <3 gaps and > = 99% identity) when BLAST conducted using the *wsp* sequences from this study, plus additional sequences of the *wsp* gene of *Wolbachia* parasitizing *P. xylostella* and *C. vestalis*.

Phylogenetic inferences were performed using the neighbor-joining (NJ) and maximum likelihood (ML) methods by PAUP*4.0b10^55^. The software MrModeltest version 2.3^56^ was used to select the best-fit nucleotide substitution model. The General Time Reversible model was used with invariable sites and a gamma-shaped distribution of rates across sites (GTR + I + G) based on the Akaike Information Criterion (AIC).

The network analysis was conducted for mitochondrial genes of *P. xylostella* and *C. vestalis* using median-joining algorithm implemented in the software Network, version 4.6.1.3^57^. We constructed the haplotype networks of both species for individual genes as well as the concatenated mitochondrial gene sequences. Haplotype type and frequency for each population were also recorded.

### Demographic analysis

We calculated the Tajima’s *D* and Fu’s *Fs* for each of the populations (≥5 individuals) of the two species based on the concatenated mitochondrial genes using Dnasp V5^49^. Analyses of mismatch distributions were also performed for both species. For *C. vestalis*, mismatch distribution was analyzed for not only a collection of all samples, but also three major clusters based on the phylogenetic tree of three concatenated mitochondrial genes.

BEAST^58^ was also used to calculate  the coalescent time of lineages in *P. xylostella* and *C. vestalis*, based on *CoxI* sequences, which has a reported mutation rate^53^. For *P. xylostella*, more samples from the Old World and Oceania^36^ were included to increase precision in coalescent time inference. As the mutation rates varied among insect lineages, we used lognormal relaxed clock while estimating the evolutionary timescales. The chain of Markov Chain Monte Carlo (MCMC) was set to 50 million with Log parameters in every 5000.

## Supplementary information

Supplementary information

## Data Availability

Data generated during the study available in Genbank with the primary accession number from KX604356 to KX606864.
