# Supplementary material for: Herbivore range expansion triggers adaptation in a subsequently-associated third trophic level species and shared microbial symbionts
Source: Sci Rep. 2019 Jul 16;9:10314. doi: 10.1038/s41598-019-46742-3 (PMC6635496; doi:10.1038/s41598-019-46742-3)
Supplement: Supplementary file 1 — Supplementary information [file 41598_2019_46742_MOESM1_ESM.doc]

**Herbivore range expansion triggers adaptation in a subsequently-associated third trophic level species and shared microbial symbionts**

Fushi Ke, Shijun You, Sumei Huang, Weijun Chen, Tiansheng Liu, Weiyi He, Dandan Xie, Qiang Li, Xijian Lin, Liette Vasseur, Geoff M Gurr and Minsheng You

**Supplementary figures and tables**

**Supplementary Figure 1.** Map Showing Sampling Locations of *P. xylostella* (•) and *C. vestalis* (•) in China, Nepal, Malaysia, Vietnam and Thailand. The inset shows the *P. xylostella* lifecycle from egg, larva, pupa to adult (left) and a female *C. vestalis* parasitizing on a third instar larva of *P. xylostella* (right)


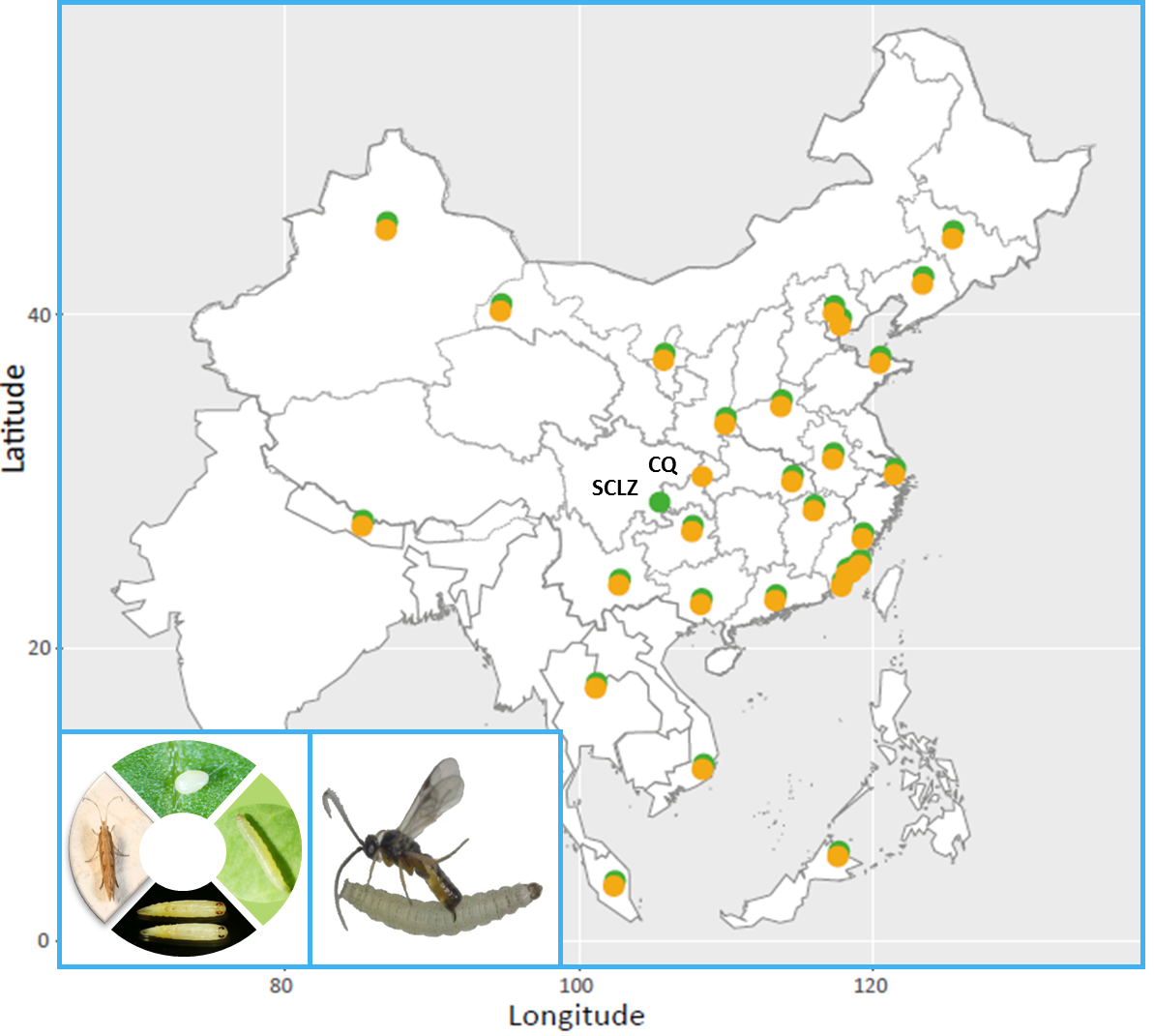


**Supplementary Table 1 Information on the *P. xylostella* and *C.* *vestalis* samples**

| **Sampling location** | **PX/CV individuals sampled** | **Latitude** | **Longitude** | **Host plants** | **Sampling time (year.month)** |
| --- | --- | --- | --- | --- | --- |
| **Shihezi, Xinjiang, China, (XJSHZ)** | 4|1 | 44.308 | 86.006 | Cabbage | 2013.8 |
| **Changchun, Jilin, China (JLCC)** | 18|14 | 43.862 | 125.326 | Cabbage | 2013.7 |
| **Shenyang, Liaoning, China (LNSY)** | 17|21 | 41.554 | 123.299 | Cauliflower and turnip | 2013.9 |
| **Jiuquan, Gansu, China (GSJQ)** | 5|1 | 40.133 | 94.649 | Cauliflower | 2012.8 |
| **Beijing, China (BJ)** | 8|4 | 40.031 | 116.279 | Turnip | 2012.1 |
| **Tianjing, China (TJ)** | 4|22 | 39.363 | 117.734 | Chinese cabbage and turnip | 2013.9 |
| **Yinchuan, Ningxia, China (NX)** | 0|1 | 38.628 | 106.066 | Cabbage | 2013.8 |
| **Zhongwei, Ningxia, China (NX)** | 1|1 | 37.475 | 105.690 | Oilseed rape | 2013.8 |
| **Qingdao, Shandong, China (SDQD)** | 19|14 | 36.306 | 120.399 | Cauliflower and cabbage | 2013.6, 2014.9 |
| **Zhengzhou, Henan, China (HNZZ)** | 20|20 | 34.868 | 113.624 | Cabbage | 2013.7 |
| **Shangluo, Shaanxi, China (SXSL)** | 8|2 | 33.870 | 109.939 | Cabbage | 2013.5 |
| **Hefei, Anhui, China (AHHF)** | 8|4 | 31.822 | 117.228 | Chinese cabbage | 2012.11 |
| **Shanghai, China (SH)** | 15|19 | 30.902 | 121.397 | Cauliflower and cabbage | 2012.10, 2014.5, 2014.9 |
| **Chongqing, China (CQ)** | 7|0 | 30.810 | 108.399 | Cabbage | 2012.1 |
| **Wuhan, Hubei, China (HBWH)** | 16|10 | 30.486 | 114.472 | Cabbage | 2014.5 |
| **Luzhou, Sichuan, China (SCLZ)** | 0|8 | 28.874 | 105.447 | Cauliflower | 2012.11 |
| **Nanchang, Jiangxi, China (JXNC)** | 14|14 | 28.723 | 115.916 | Chinese cabbage | 2014.6 |
| **Guiyang, Guizhou, China (GZGY)** | 17|12 | 26.458 | 106.600 | Cabbage | 2012.1 |
| **Fuzhou, Fujian, China (FJFZ)** | 13|19 | 26.010 | 119.238 | Cauliflower and Chinese cabbage | 2014.3 |
| **Putian, Fujian, China (FJPT)** | 8|3 | 25.359 | 119.041 | Cauliflower and Cabbage | 2013.11 |
| **Quanzhou, Fujian, China (FJQZ)** | 11|10 | 24.922 | 118.517 | Cauliflower and Cabbage | 2013.12 |
| **Xiamen, Fujian, China (FJXM)** | 7|14 | 24.681 | 118.139 | Cauliflower and Cabbage | 2013.12 |
| **Yuxi, Yunnan, China (YNYX)** | 15|14 | 24.109 | 102.758 | Cauliflower and Cabbage | 2012.11, 2014.6 |
| **Zhangzhou, Fujian, China (FJZZ)** | 7|3 | 24.036 | 117.815 | Cabbage | 2013.12 |
| **Guangzhou, Guangdong, China (GDGZ)** | 17|17 | 23.123 | 113.332 | Cauliflower and Cabbage | 2012.11, 2014.6 |
| **Nanning, Guangxi, China (GXNN)** | 14|19 | 22.862 | 108.301 | Chinese cabbage | 2012.1 |
| **Katmandu, Nepal（NPKT）** | 7|17 | 27.685 | 85.365 | Cabbage | 2013.9 |
| **Phetchabun, Thailand (TLPH)** | 7|10 | 16.417 | 101.190 | Cabbage | 2013.7 |
| **Dalat, Vietnam (VTDL)** | 14|14 | 11.958 | 108.420 | Cauliflower and Cabbage | 2013.8 |
| **Kota Kinabalu, Malaysia (MLKK)** | 12|10 | 5.9843 | 116.576 | Cabbage | 2013.1 |
| **Cameron highland, Malaysia (MLCH）** | 11|13 | 3.9380 | 102.420 | Cauliflower | 2013.11 |

**Note:** PX and CV represent *P. xylostella* and *C. vestalis*, respectively. We combined samples from Yinchuan, Ningxia, China and Zhongwei, Ningxia, China into one population for analysis.

**Supplementary Table 2 Information on the gene fragments and related primers used in *P. xylostella*, *C. vestalis* and *Wolbachia***

| **Taxon** | **Gene** | **Primers** | **Primer sequences (5’-3’)** | **Annealing temperature (℃)** | **Fragment length (bp)** | **Source** |
| --- | --- | --- | --- | --- | --- | --- |
| *P. xylostella* | *CoxI* | DBM*-coxI*-F | AAATTTACAATTTATCGCTTAATCTCAGCC | 55 | 800-1000 | Yukuhiro *et al*., 1994 |
|  |  | DBM*-coxI*-R | CCTTTTCTTGTGTAATAATATGGAAATTATACC |  |  | Yukuhiro *et al*., 1994 |
|  | *Cytb* | DBM*-cytb-*2F | ACACGCTAATGGAGCATC | 60 | 550-600 | This study |
|  |  | DBM*-cytb*-2R | CTGGTTGAATGTGAATAGGA |  |  | This study |
|  | *NadhI* | DBM*-nadhI*-2F | ATCATAACGATAACGAGG | 55 | 730-770 | This study |
|  |  | DBM*-nadhI*-2R | CAAATTCGTAAAGGTCCT |  |  | This study |
| *C. vestalis* | *CoxI* | CV*-coxI*-F | GGTCAACAAATCATA AAGATATTGG | 58 | 650-700 | Folmer *et al*., 1994 |
|  |  | CV*-coxI*-R | TAAACTTCAGGGTGACCAAAAATCA |  |  | Folmer *et al*., 1994 |
|  | *Cytb* | CV*-cytb*-F | TATGTACTACCATGAGGACAAATATC | 50 | 460-500 | Simon *et al*., 1994 |
|  |  | CV*-cytb*-R | ATTACACCTCCTAATTTATTAGGAAT |  |  | Simon *et al*., 1994 |
|  |  | CV*-cytb*-2F | CGAACTACCAACACCAATTA | 58 | 900-1000 | This study |
|  |  | CV*-cytb*-2R | TGGGTATTCTACAGGTTGAG |  |  | This study |
|  | *NadhI* | CV*-nadhI*-F | ACTAATTCAGATTCTCCTTCT | 50 | 460-500 | Smith & Kambhampati, 1999 |
|  |  | CV*-nadhI*-R | CAACCTTTTAGTGATGC |  |  | Smith *et al*., 1999 |
|  |  | CV-*nadhI*-5F | TTCGAGGCAAAGTTATTC | 55 | 700-750 | This study |
|  |  | CV-*nadhI*-7R | ATTATCGGAAAGGACCTA |  |  | This study |
| *Wolbachia* | *wsp* | *wsp*81F | TGGTCCAATAAGTGATGAAGAAAC | 50.5 | 550-600 | Braig *et al*., 1998 |
|  |  | *wsp*691R | AAAAATTAAACGCTACTCCA |  |  | Braig *et al*., 1998 |

**Supplementary References**

Yukuhiro, K., Sezutsu, H., Itoh, M., Shimizu, K. and Banno, Y. 2002. Significant levels of sequence divergence and gene rearrangements have occurred between the mitochondrial genomes of the wild mulberry silkmoth, *Bombyx mandarina*, and its close relative, the domesticated silkmoth, *Bombyx mori*. *Mol. Biol. Evol*. **19**, 1385-9.

Folmer, O., Black, M., Hoeh, W., Lutz, R. & Vrijenhoek, R. 1994. DNA primers for amplification of mitochondrial cytochrome c oxidase subunit I from diverse metazoan invertebrates. *Mol. Mar. Biol. Biotechnol.* **3**, 294-299.

Simon, C. 1994. Evolution, weighting, and phylogenetic utility of mitochondrial gene sequences and a compilation of conserved polymerase chain reaction primers. Ann. Entomol. Soc. Am. *87*, 651-701.

Smith, P.T. & Kambhampati, S. 1999. Status of the *Cotesia flavipes* species complex (Braconidae: Microgastrinae) based on mitochondrial *16S rRNA* and *NADH 1* dehydrogenase gene sequence. *J. Kansas Entomol. Soc.* **72**, 306-314.

Smith, P.T., Kambhampati, S., Völkl, W. & Mackauer, M. 1999. A phylogeny of aphid parasitoids (Hymenoptera: Braconidae: Aphidiinae) inferred from mitochondrial *NADH 1* dehydrogenase gene sequence. *Mol. Phylogenet. Evol.* **11**, 236-245.

Braig, H.R., Zhou, W., Dobson, S.L. & O Neill, S.L. 1998. Cloning and characterization of a gene encoding the major surface protein of the bacterial endosymbiont *Wolbachia pipientis*. *J. Bacteriol.* **180**, 2373.
